# Neonatal Exposure to Di(2-ethylhexyl) Phthalate Is Associated with Lung Injury in a Rat Model of Chronic Lung Disease of Prematurity

**DOI:** 10.3390/toxics14060517

**Published:** 2026-06-12

**Authors:** Shahana Perveen, Li Lou, Sohini Alim, Abigail Akselrod, Chunfang Zhao, Namita Sen, Clifford S. Deutschman, Annemarie Stroustrup

**Affiliations:** 1 Northwell Health, New Hyde Park, NY 11040, USA; llou@northwell.edu (L.L.); sohinialim@gmail.com (S.A.); abbyakselrod1@gmail.com (A.A.); czhao@northwell.edu (C.Z.); nsen@northwell.edu (N.S.); cdeutschman@northwell.edu (C.S.D.); astroustrup@northwell.edu (A.S.); 2 Division of Neonatology, Cohen Children’s Medical Center, New Hyde Park, NY 11040, USA; 3 Department of Pediatrics, Zucker School of Medicine at Hofstra/Northwell, Hempstead, NY 11549, USA; 4 Institute for Molecular Medicine, Feinstein Institutes for Medical Research, Manhasset, NY 11030, USA; 5 Department of Occupational Medicine, Epidemiology & Prevention, Zucker School of Medicine at Hofstra/Northwell, Hempstead, NY 11549, USA; 6 Institute for Health System Science, Feinstein Institutes for Medical Research, Manhasset, NY 11030, USA

**Keywords:** phthalates, environmental toxin, bronchopulmonary dysplasia, chronic lung disease, lung injury, prematurity, animal model

## Abstract

Chronic lung disease of prematurity (CLD) is a common complication of preterm birth with a complex pathology. Recent epidemiologic studies have identified a link between neonatal exposure to di(2-ethylhexyl) phthalate (DEHP), frequently used in medical equipment, and the development of CLD. We hypothesize that DEHP exposure in the early neonatal period contributes to lung injury in newborn rats. Newborn rat pups were raised in one of the following environments: room air (RA), RA + DEHP, hyperoxia (60% oxygen), and hyperoxia + DEHP. Ambient DEHP was inhaled at a dose of 25 mg/m^3^ for 6 h daily for 14 days. Lung tissue and blood samples were collected on the 14th day of life. Independent exposure to DEHP and hyperoxia resulted in thicker pulmonary septal walls, fewer alveoli, increased pulmonary polymorphonuclear leukocytes and myeloperoxidase (MPO) activity and decreased expression of CD31 on endothelial cells in lung tissue. Additionally, DEHP-exposed rats showed higher serum malondialdehyde (MDA) levels and reduced vascular endothelial growth factor (VEGF) mRNA and protein levels compared to controls. Our experiments demonstrate that inhaled DEHP, with or without hyperoxia, resulted in a similar pattern of morphological lung injury and inflammation characteristic of CLD, suggesting an association with CLD of prematurity.

## 1. Introduction

Preterm birth is associated with significant morbidity and mortality [1], largely attributed to the disruption of second- and third-trimester development of multiple organs, prominently the lungs. Chronic lung disease related to prematurity (CLD), also referred to as bronchopulmonary dysplasia (BPD), is a multifactorial disorder. The development of CLD in preterm infants has been linked to volutrauma and barotrauma due to mechanical ventilation [2], infection and non-infectious inflammation [3], and exposure to hyperoxia [2,3,4]. However, these factors alone do not predict the development of CLD with high sensitivity or specificity [5,6,7,8]. It is therefore likely that other factors contribute to, and perhaps even precipitate, CLD of prematurity. As improvements in neonatal intensive care continue to reduce known contributors to CLD (barotrauma, volutrauma, infection-driven inflammation) without significantly decreasing disease incidence [9], a search for additional modifiable factors that contribute to CLD pathobiology is imperative.

A recent epidemiologic study identified an association between NICU-based exposure to inhaled environmental phthalates and the development of CLD in a large, multi-site cohort of preterm infants [10]. Phthalate diesters, the most ubiquitous of which is di(2-ethylhexyl) phthalate (DEHP), are commonly used organic chemical adjuvants that make plastic materials soft and pliable [11,12]. Exposure to specific phthalates via the respiratory circuit is associated with adverse clinical outcomes in preterm infants [13]. Both in vitro and animal studies indicate that phthalates activate inflammation and dysregulate cellular pathways [14,15] that have been implicated in the development of CLD [16,17,18]. The contribution of phthalate exposure via inhalation to the pathogenesis and pathobiology of CLD has not been specifically examined. Prior studies in a rat model have demonstrated that DEHP exposure through various routes, including intravenous and gavage feeding during gestation and early life, correlates with adverse outcomes including overall poor growth, delayed lung development, impaired cell proliferation, and histological abnormalities in lung tissue [19,20]. Preterm infants requiring respiratory support are commonly exposed to significant levels of DEHP daily during early life, primarily via inhalation. The inhalation route of exposure is biologically distinct from enteral or intravenous routes due to differences in local tissue deposition, vulnerability of the developing lung and differences in first-pass metabolism. However, its specific role in the pathogenesis of chronic lung disease of prematurity remains largely unexplored.

To address this gap in knowledge we designed a system that delivers aerosolized DEHP to neonatal rats in a manner that mimics the inhalational exposure experienced by preterm infants receiving respiratory support. Unlike the clinical correlate, our free breathing animal model is not confounded by barotrauma or volutrauma from positive pressure respiratory support, a significant step forward in the study of the impact of phthalate exposure on lung development. In the data detailed here, we report on the use of this system to investigate the contribution of DEHP, administered with or without supplemental oxygen, to the pathogenesis and mechanistic pathobiology of neonatal lung injury in a rat model of CLD.

## 2. Materials and Methods

All procedures and protocols were approved by the Institutional Animal Care and Use Committees at The Feinstein Institutes for Medical Research (Protocol #24-0631) and conformed to ARRIVE guidelines [21,22].

### 2.1. Animal Model

We adapted an established model of CLD due to prematurity in which a sealed chamber enables continuous delivery of specific oxygen concentrations to induce CLD in newborn rat pups [23]. Our modified chamber allows for the introduction of aerosolized DEHP. The system, which contains two separate cages for rat pups and their mother, is equipped with a measurement device “Micro Dust Pro” for DEHP concentration monitoring (CH Technologies, Westwood, NJ, USA), and an AMI oxygen analyzer to measure oxygen concentrations in the ambient air (Advanced Micro Instruments Inc 225, Paularino Avenue, Costa Mesa, CA 92626, USA).

### 2.2. DEHP Preparation

DEHP (CAS No. 117-81-7, 0.985 g/mL, Sigma-Aldrich Corp., St. Louis, MO, USA) was passed through an aerosol generator (4-jet refillable Blam nebulizer, CH Technologies, Westwood, NJ, USA) and continuously administered.

Based on a prior study [24] that demonstrated the toxic effects of DEHP in rats, we chose an inhalation dose of 25 mg/m^3^ of DEHP to study its effect on newborn rat lung. Preterm infant exposure to DEHP varies widely based on duration and type of respiratory support. Cumulative exposure of DEHP can vary from 29 to 123 µg and may reach 16 mg/kg/day in critically ill preterm infants. Exposure to 25 mg/m^3^ DEHP via inhalation translates to approximately 7.2 mg/kg/day, well within the range of clinically relevant exposure levels. For comparison, the upper limit of exposure for adult workers is 5 mg/m^3^, as recommended by OSHA.

### 2.3. Experimental Conditions

Pregnant female Sprague Dawley rats (Charles River, Wilmington, MA, USA) were maintained in a biosafety level 2 (BSL2) room at the animal facility of the Feinstein Institutes for Medical Research. Normal day/night light cycles were maintained. After delivery and recovery, the mother and newborn rat pups were allocated to one of four groups. Pups and dams were maintained in the chamber for 14 days. Animals were exposed to an ambient air mixture, as specified in Table 1. On day 14, all pups were euthanized; blood was obtained by cardiac puncture, and serum was isolated and stored at −80 °C. The lungs were extracted en bloc, lung tissue was homogenized, and RNA and protein fractions were isolated as described below. For histopathology, the lungs were inflated and fixed with a 1:1 mixture of 4% paraformaldehyde in phosphate-buffered saline and optimal cutting temperature compound (OCT). This solution was infused through the trachea, using a fixed volume of 1 mL, as a slow infusion over 2–3 min. Inflation was volume-dependent and not pressure-dependent. We did not use constant pressure to avoid barotrauma on the newborn lung. Using a fixed volume with a slow infusion rate is an acceptable method for lung inflation for histopathological evaluations [25]. The right lung was embedded in paraffin and cut into 6 µm slices. Lung sections were stained with hematoxylin and eosin (H&E). The left lung was frozen and cryosectioned into 10 µm slices for immunofluorescence staining (IF).

### 2.4. Histological Preparation

Paraffin-embedded H&E-stained lung sections were examined under light microscopy (Zeiss Axio observer 7, White Plains, NY, USA). For each animal, non-contiguous fields were evaluated on representative slides to determine average septal thickness and polymorphonuclear cell (PMN) count at 40× magnification, and radial alveolar count (RAC) at 10× magnification. Histological evaluations were performed independently by three researchers who were partially blinded using standardized criteria.

#### 2.4.1. Septal Thickness

The mean thickness of 100 septa on ten non-contiguous 40× fields per animal was determined as previously described [26].

#### 2.4.2. Radial Alveolar Count (RAC)

**Figure A1:**
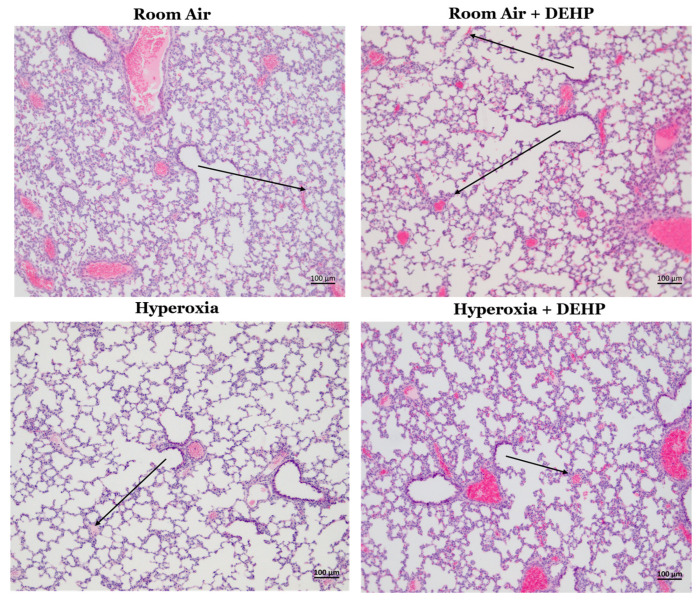
Representative 10× magnification fields showing lines measuring radial alveolar count (RAC) in neonatal rat lungs in room air and following inhaled DEHP, hyperoxia, or hyperoxia + DEHP. Neonatal Sprague Dawley rats breathing the mixture were observed for 14 days. Lines connect a terminal bronchiole to the nearest acinar edge, with alveolar intersections counted to determine RAC.

RAC, an index of alveologenesis [27,28], was determined by (1) identifying a terminal respiratory bronchiole, (2) drawing a vertical line from the center of a terminal respiratory bronchiole to the nearest acinar edge (either intra-alveolar septum or pleura), and (3) counting the number of alveoli cut by this line. A sample measurement can be found in Appendix A Figure A1. Radial alveolar count was measured under 10× magnification by averaging the count from ten or fewer randomly chosen fields per animal.

#### 2.4.3. Polymorphonuclear (PMN) Count

Each non-contiguous 40× field was divided into a 3 × 3 grid. The number of PMN cells in each of the nine grid spaces was counted, and the mean was taken for the field. Means from 10 such fields per slide were used to generate PMN counts for the entire lung. Images were analyzed using ImageJ software 2.1/image J2 (National Institutes of Health).

### 2.5. Immunofluorescence Preparation

Cryosections of lung tissues were stained by immunofluorescence for target antigens and were counterstained with 4′6-diamidino-2-phenylindole (DAPI) mounting media (Vector Laboratories, Newark, CA, USA, H-1200-10) for nuclear visualization. For each target, non-overlapping fields of view were randomly acquired using a confocal microscope (Zeiss, White Plains, NY, USA, LSM880). Confocal settings (gain, laser power, and pinhole size) were optimized for each individual target and then held constant for all subsequent imaging of that target. Target-positive area was quantified by thresholding using ImageJ2 software (National Institutes of Health). For each target, the threshold was determined from no-secondary antibody control and subsequently held constant for all images analyzed. Target-positive cells were counted and identified as DAPI-positive nuclei colocalizing with the target-positive signal. The target-positive area per target-positive cell was then calculated for each image. For each animal, values from all acquired images were averaged for statistical analysis.

#### 2.5.1. Myeloperoxidase (MPO)

For myeloperoxidase (MPO) staining, cryosections were incubated with Rabbit Anti-MPO primary antibody (ABCAM, Cambridge, UK, AB208670, 1:200) followed by an Alexa Fluor Plus 594 conjugated goat anti-rabbit secondary antibody (Invitrogen, Waltham, MA, USA, A3653401, 1:500). Ten random non-overlapping fields of view were acquired per rat at 20× magnification. MPO expression was defined as the mean ratio of MPO-positive area per MPO-positive cell for all fields of view per animal.

#### 2.5.2. CD31/PECAM-1

For platelet endothelial cell adhesion molecule-1 (PECAM-1/CD31) staining, cryosections were incubated with Goat anti-CD31 primary antibody (R&D Systems, Minneapolis, MN, USA, AF3628, 1:250) followed by an Alexa Fluor 594 conjugated donkey anti-goat secondary antibody (Invitrogen, Waltham, MA, USA, A11058, 1:500). Three to eight random non-overlapping fields of view were acquired per rat at 40× magnification. Prior to quantification, mean intensity projections were generated from full z-stacks for each field using Zen Black software v2.3 (Carl Zeiss, White Plains, NY, USA). CD31 expression was defined as the mean ratio of CD31-positive area per CD31-positive cell for all fields of view per animal.

### 2.6. RNA Isolation and cDNA Synthesis

Total RNA was isolated from a sample of lung homogenate weighing 20–30 mg (RNeasy Mini Kit, Cat. 74104, Qiagen, Germantown, MD, USA) per the manufacturer’s instructions. Concentration was determined spectrophotometrically (Nanodrop Technologies, Wilmington, DE, USA). A 260/280 ratio of 2.0 was accepted as “pure” for RNA [29]. Complementary DNA (cDNA) was created from total RNA (1 µg) via reverse transcription (iScript gDNA Clear cDNA Synthesis Kit, Bio-Rad Life Science, Cat. 1725034, Hercules, CA, USA) per the manufacturer’s instructions. Concentration was determined spectrophotometrically. A 260/230 ratio of 1.8 was accepted as “pure” for DNA [29].

### 2.7. RT-PCR

RT-PCR was performed using TaqMan technology (TaqMan 7900HT Fast Real-Time PCR System, Applied Biosystems, Carlsbad, CA, USA) per the manufacturer’s instructions. PCR primers and probes for VEGF (Rn01511602_m1) and β-actin (Rn00667869_m1) were obtained from Thermo Fisher Scientific (Waltham, MA, USA). Both template cDNA (positive) and/or RNase-free water (negative) served as controls. The PCR cycling protocol used involved 40 cycles of (1) denaturing (95 °C for 10 s) and (2) primer annealing and extension (60 °C for 1 min), followed by fluorescent labeling. The relative gene expression was calculated according to the comparative C_t_ method (2^−ΔΔC^_t_ or ΔΔC_t_ method) [30]. cDNA concentration was normalized to the concentration of β-actin cDNA in the same sample.

### 2.8. Western Blotting

Total protein was extracted from frozen lung homogenate via radioimmunoprecipitation (Lysis Buffer System, Cat. sc-24948, Santa Cruz Biotechnology, Inc., Dallas, TX, USA) in the presence of phosphatase (Pierce, Cat. PIA32957, Thermo Fisher Scientific, Waltham, MA, USA) and protease (Pierce, Cat. PIA32955, Thermo Fisher Scientific, Waltham, MA, USA) inhibitors. Total concentration was determined using the BCA Protein Assay Kit (Thermo Fisher Scientific, Cat. PI23225, Waltham, MA, USA) per the manufacturer’s instructions. Then, 20 µg of denatured protein was suspended in 12 µL total volume (water + sample loading buffer) and subjected to sodium dodecyl sulfate–polyacrylamide gel electrophoresis (SDS-PAGE) on a gradient (4–15%) precast gel (Mini-PROTEAN^®^ TGX™ Precast Protein Gels, Cat. 4561084, Bio-Rad Life Science, Hercules, CA, USA) at 150 V for 45 min. The gel was transferred onto polyvinylidene fluoride (PVDF) membranes and blocked with bovine serum albumin for 1 h. Membranes were then incubated overnight at 4 °C with primary monoclonal antibodies to VEGF (Novus Biologicals, Cat. NB100-664, Centennial, CO, USA, diluted 1:1000), VEGFR1 (Abcam, Cat. ab32152, Waltham, MA, USA, diluted 1:2000), or VEGFR2 (Abcam, Cat. ab221679, Waltham, MA, USA, diluted 1:2000). β-actin (Abcam, Cat. mAbcam8226, Waltham, MA, USA, diluted 1:1000) was also determined as a loading control. Membranes were treated with secondary detection antibodies: goat anti-mouse immunoglobulin G (IgG) (Abcam, Cat. ab205719, Waltham, MA, USA) diluted 1:2000 or goat anti-rabbit IgG (Abcam, Cat. ab205718, Waltham, MA, USA) diluted 1:2000 and subjected to detection via enhanced chemiluminescence (Thermo Fisher Scientific, Cat. 32106, Waltham, MA, USA). The intensities of protein bands were quantified by Image Lab software 6.1 (Bio-Rad, Hercules, CA, USA). Each homogenate sample was assessed in triplicate; if an individual value differed from the other two by >33%, the process was repeated. Mean values from each individual animal were used to determine the mean and standard deviation (SD) for the group.

### 2.9. ELISA

ELISA determination of MDA (Abcam, Cat. ab238537, Waltham, MA, USA) levels in serum were performed per manufacturer instructions.

### 2.10. Statistical Analysis

All data are presented as the mean ± standard deviation (SD). Statistical analyses were performed using GraphPad Prism 9 software (GraphPad Software, San Diego, CA, USA). Normality of distribution of residuals was assessed using D’Agostino–Pearson omnibus, Anderson–Darling, Shapiro–Wilk, and Kolmogorov–Smirnov tests and by examination of Q:Q plots. If residuals were normally distributed, we applied one-way analysis of variance (ANOVA) followed by the Tukey correction for multiple comparisons (*p* ≤ 0.05).

## 3. Results

**Figure A2:**
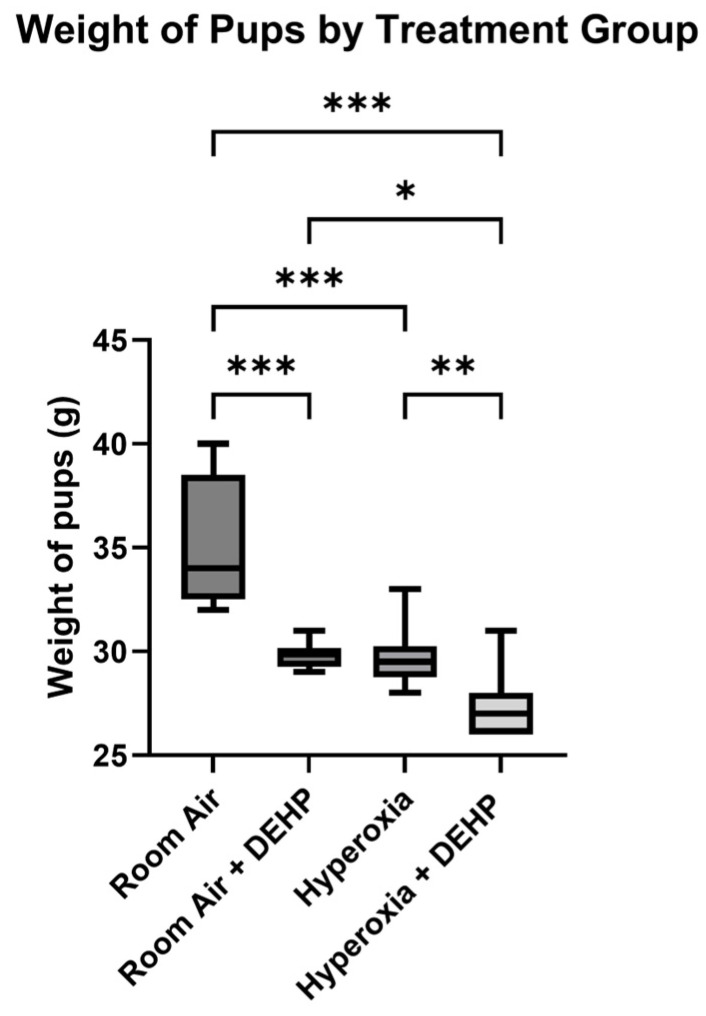
Weight of neonatal rat pups in room air and following inhalation of DEHP, hyperoxia, or hyperoxia + DEHP. Neonatal Sprague Dawley rats breathing the mixture were observed for 14 days. n = 8–14 animals/intervention. Data is shown as a box and whisker plot with whiskers indicating the minimum and maximum values in each group. Significance is determined using one-way ANOVA with Tukey’s correction. * *p* ≤ 0.05, ** *p* ≤ 0.01, *** *p* ≤ 0.001.

We studied seven dams and their offspring. Pup numbers per experimental group were as follows: room air (RA) (n = 13), DEHP (n = 8), hyperoxia (n = 14), and hyperoxia + DEHP (n = 14). A significant reduction in average body weight (*p* ≤ 0.0001) was observed on the 14th day of life in the DEHP, hyperoxia and hyperoxia + DEHP groups compared to the RA (Figure A2 and Table A1). Combined exposure to hyperoxia + DEHP resulted in the largest decrement in body weight.

### 3.1. Morphologic Lung Injury

**Figure 1:**
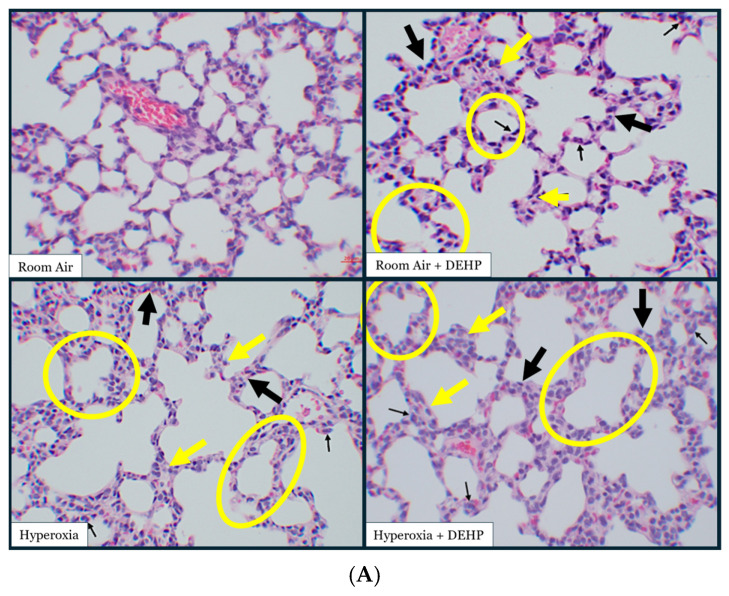

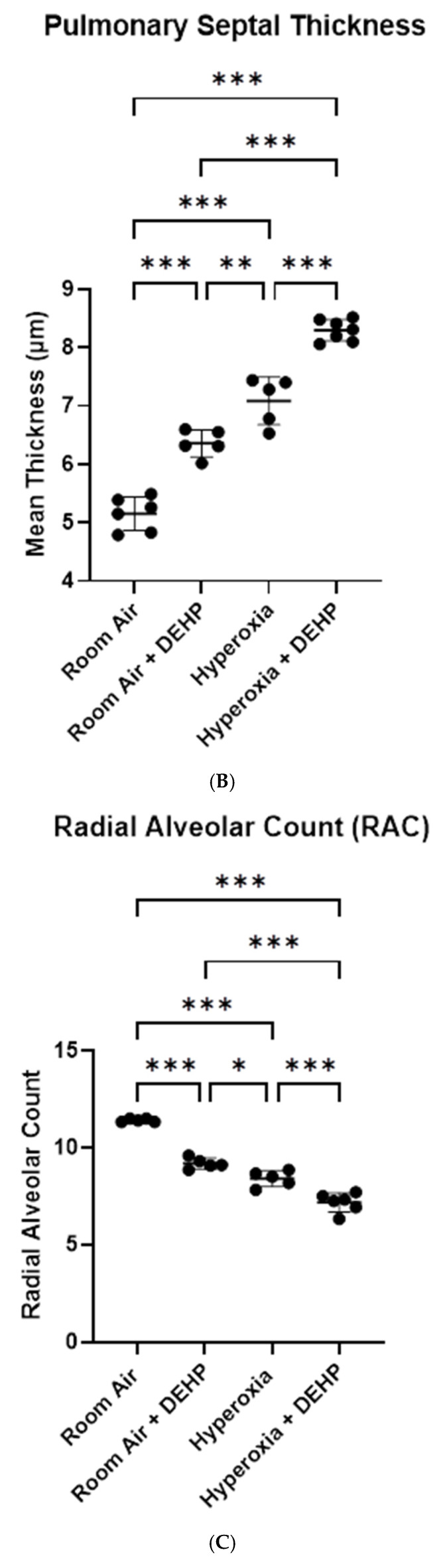
(**A**) Responses of neonatal lungs in room air and following inhaled DEHP, hyperoxia, and hyperoxia + DEHP. Representative H&E-stained sections, 40× magnification. Neonatal Sprague Dawley rats breathing the mixture were observed for 14 days. Description in the text. Arrows—neutrophils. (**B**) Septal thickness in neonatal rat lungs in room air and following inhaled DEHP, hyperoxia, or hyperoxia + DEHP. Neonatal Sprague Dawley rats breathing the mixture were observed for 14 days. Y-axis—mean thickness in µm. Measurements were averaged from counts on 10 non-contiguous 40× magnification fields/animal; n = 5–7 animals/intervention. Data is shown as Mean ± SD. Significance was determined using one-way ANOVA with Tukey’s correction. ** = *p* ≤ 0.01, *** = *p* ≤ 0.001. (**C**) Radial alveolar count (RAC) in neonatal rat lungs in room air and following inhaled DEHP, hyperoxia, or hyperoxia + DEHP. Neonatal Sprague Dawley rats breathing the mixture were observed for 14 days. Y-axis—number of alveoli crossed by a line connecting a terminal bronchiole to the nearest acinar edge. Measurements were averaged from counts on 10 non-contiguous 10× magnification fields/animal; n = 5–6 animals/intervention. Data is shown as Mean ± SD. Significance was determined using one-way ANOVA with Tukey’s correction. * = *p* ≤ 0.05, *** = *p* ≤ 0.001.

Representative H&E-stained sections from animals in each of the four groups are provided in Figure 1A. These images qualitatively demonstrate the key features of lung injury: distorted, malformed (yellow circle) or absent alveoli; septal edema/thickening (black arrow); and an interstitial PMN infiltrate (yellow arrows).

Measurements of septal thickness are quantified in Figure 1B. This data demonstrates that septal thickness was significantly greater than RA control group following exposure to either DEHP or hyperoxia alone (*p* ≤ 0.001). Septal thickness in rats exposed to hyperoxia + DEHP, in turn, was significantly greater than RA (*p* ≤ 0.001) and was also more pronounced than the difference detected following exposure to each individual agent (*p* ≤ 0.001). Indeed, the increase in mean septal thickness after exposure to both agents was roughly equal to the sum of increase over the baseline RA group of septa exposed to each individual agent in isolation.

Figure 1C details the quantification of RAC, a measure of alveolar development [28,31]. RAC declined significantly following exposure to either DEHP or hyperoxia alone (*p* ≤ 0.001). Once again, the difference from RA following exposure to a combination of hyperoxia and DEHP was roughly equal to the sum of the differences from RA control for each individual agent. This data suggests that both DEHP and hyperoxia alone induce lung injury. The summative nature of the degree of injury caused by the two in combination, as opposed to either less profound damage or a synergistic response, suggests an element of independence in the underlying mechanisms.

### 3.2. Inflammation and Oxidative Stress

**Figure 2:**
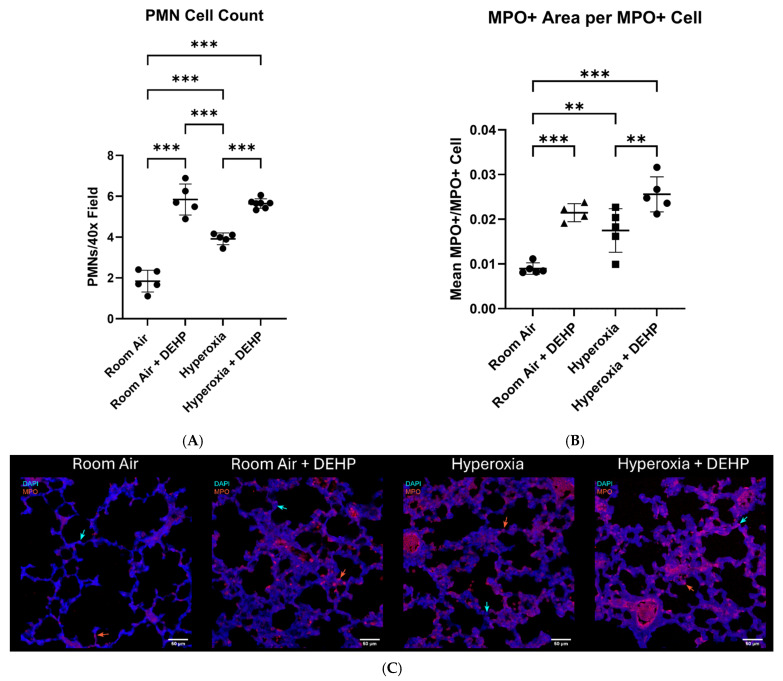
(**A**) Mean number of PMNs/40× field in neonatal rat lungs in room air and following inhaled DEHP, hyperoxia, or hyperoxia + DEHP. Neonatal Sprague Dawley rats breathing the mixture were observed for 14 days. Measurements were obtained as described in Section 2. n = 5–7 animals/intervention. Data is shown as Mean ± SD. Significance was determined using one-way ANOVA with Tukey’s correction. *** = *p* ≤ 0.0001. (**B**) Mean MPO+ Area/MPO+ Cell in neonatal rat lungs in room air and following inhaled DEHP, hyperoxia, or hyperoxia + DEHP. Neonatal Sprague Dawley rats breathing the mixture were observed for 14 days. Y-axis—mean area of MPO+ immunofluorescence staining per MPO+ cell (myeloperoxidase-positive neutrophils). Measurements were obtained as described in Section 2; n = 4–5 animals/intervention. Data is shown as Mean ± SD. Significance was determined using one-way ANOVA with Tukey’s correction. ** = *p* ≤ 0.01, *** = *p* ≤ 0.001. (**C**) Representative 20× fields of MPO and DAPI staining in neonatal rat lungs in room air and following inhaled DEHP, hyperoxia, or hyperoxia + DEHP.

Data assessing the effects of DEHP, hyperoxia, or hyperoxia + DEHP on PMN accumulation are depicted in Figure 2A. We found that the number of PMNs/40× magnification field was higher than the RA control group under all three experimental conditions. There were more PMNs following DEHP exposure than following hyperoxia. PMN counts after exposure to both hyperoxia and DEHP, while significantly higher than those noted following hyperoxia alone, could not be distinguished from numbers determined following exposure to DEHP alone. These findings suggest that PMN accumulation after either hyperoxia or DEHP involves a similar underlying mechanism.

MPO is a heme-containing peroxidase enzyme present inside the neutrophil. Representative sections showing MPO are depicted in Figure 2B,C, confirming PMN presence in the lung tissue. Results showed a significant increase in MPO expression in the DEHP group compared to the RA group (*p* ≤ 0.001). The hyperoxia group also showed an increase in MPO expression, but the effect was less pronounced than the DEHP-alone group (*p* ≤ 0.01). Exposure to both hyperoxia and DEHP again showed a significant increase in MPO expression compared to the RA control group (*p* ≤ 0.001).

**Figure 3:**
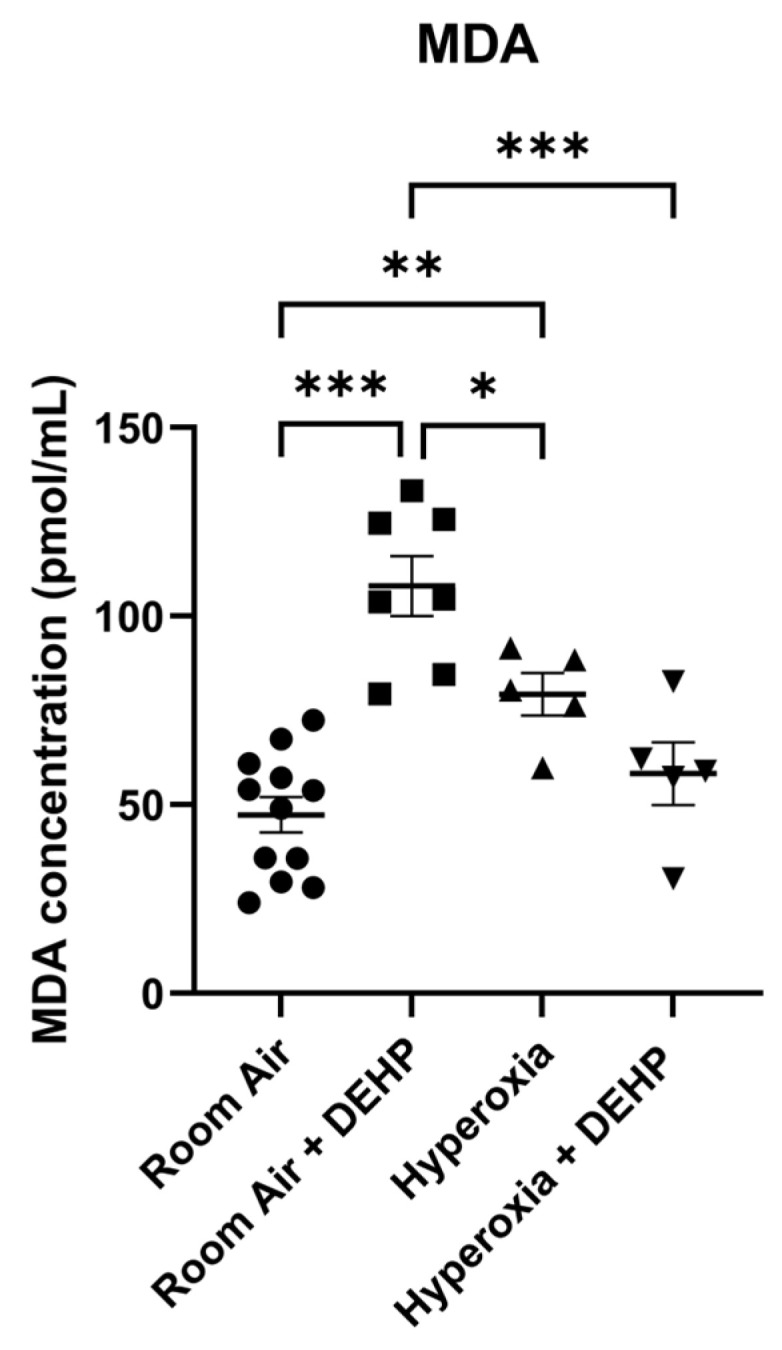
Malondialdehyde (MDA) concentration in serum. Neonatal Sprague Dawley rats breathing the mixture were observed for 14 days. Measurements were obtained as described in Section 2. n = 5–12 animals/intervention. Data is shown as Mean ± SD. Significance was determined using one-way ANOVA with Tukey’s correction. * = *p* ≤ 0.05, ** = *p* ≤ 0.01, *** = *p* ≤ 0.001.

MPO released by activated PMNs serves as a critical mechanistic bridge linking inflammation to oxidative stress, with MDA functioning as a downstream biomarker of lipid peroxidation that confirms this transition. Thus, MDA concentration in the blood has been used as a marker of oxidative injury [32,33]. Serum levels of MDA in our three experimental groups are graphically depicted in Figure 3. This data shows that DEHP, hyperoxia, and hyperoxia + DEHP increased the serum MDA concentration compared to RA.

### 3.3. Vascular Injury

**Figure 4:**
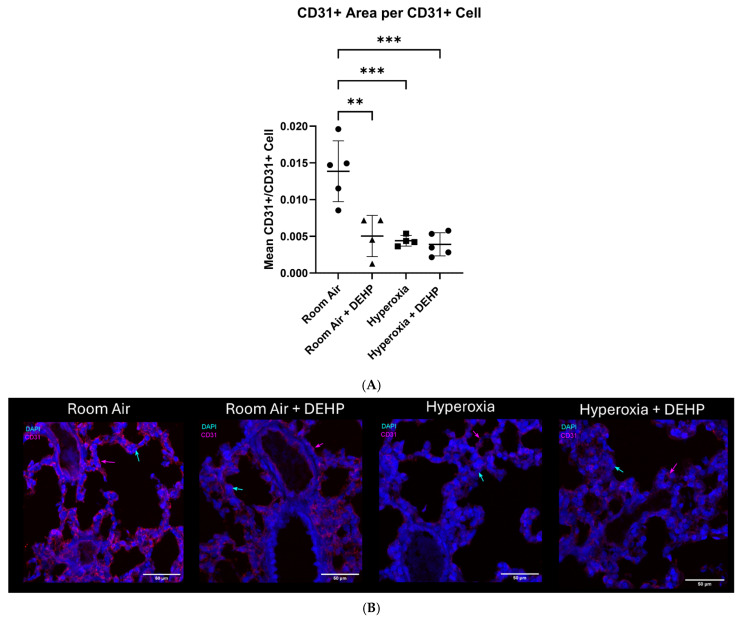
(**A**) Mean CD31+ Area/CD31+ Cell in neonatal rat lungs at baseline and following inhaled DEHP, hyperoxia, or hyperoxia + DEHP. Neonatal Sprague Dawley rats breathing the mixture were observed for 14 days. Y-axis—mean area of CD31+ immunofluorescence staining per CD31+ cell. Measurements were obtained as described in Section 2; n = 4–5 animals/intervention. Data is shown as Mean ± SD. Significance was determined using one-way ANOVA with Tukey’s correction. ** = *p* ≤ 0.01, *** = *p* ≤ 0.001. (**B**) Representative 40× mean intensity projections of CD31 and DAPI staining in neonatal rat lungs in room air and following inhaled DEHP, hyperoxia, or hyperoxia + DEHP.

One prominent characteristic of CLD is a reduced and dysmorphic vascular bed [34]. CD31/PECAM-1 is highly expressed at endothelial cell intercellular junctions and vascular cells and is best known as a marker for the vascular endothelium to identify blood vessels. The expression of CD31 in the DEHP group was significantly decreased relative to RA control (*p* ≤ 0.01). CD31 expression after exposure to hyperoxia and hyperoxia + DEHP was lower (*p* ≤ 0.001) than in the RA control group (Figure 4A,B).

**Figure 5:**
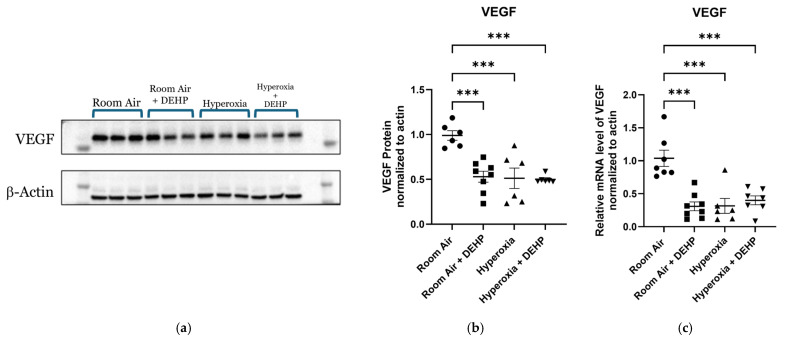
Relative abundance of VEGF in lung homogenate. Neonatal Sprague Dawley rats breathing the mixture were observed for 14 days. Data is shown as Mean ± SD. Significance was determined using one-way ANOVA with Tukey’s correction. *** = *p* ≤ 0.001. (**a**) Representative immunoblot. Signal generated by VEGF normalized to signal generated by β-actin in the same sample. Intensity from each animal was assessed in triplicate; the mean of these was used for quantification. (**b**) Quantification of fluorescence intensity on immunoblots. Mean signals from each animal were used to generate Mean ± SD for each group. n = 6–8 animals/intervention. (**c**) Data generated using RT-PCR as described in Section 2. Intensity was determined using the ΔΔCt method normalized to signal from mRNA encoding β-Actin in the same sample. n = 6–8 animals/intervention.

Vascular endothelial growth factor (VEGF) is a potent contributor to angiogenesis during embryonic development [35] and has been implicated in the pathogenesis of hyperoxic lung injury [36]. We first examined VEGF abundance in lung homogenate. Figure 5a depicts a representative immunoblot of VEGF and a β-actin loading control, while quantification is presented in Figure 5b. This data indicates that the abundance of VEGF protein is significantly less than the RA control group following exposure to either hyperoxia or DEHP. Levels post exposure to hyperoxia + DEHP were significantly lower than baseline but were indistinguishable from values of either agent alone. Because VEGF levels are in part transcriptionally controlled [37], we assessed levels of the mRNA encoding VEGF in our four exposure groups (Figure 5c). Abundance in all three intervention groups was significantly lower than abundance at RA control group. These findings suggest that intervention interferes with VEGF transcription or mRNA degradation. The effects of exposure to hyperoxia + DEHP do not indicate an additive or synergistic process. These findings suggest that overlapping mechanisms lower DEHP and hyperoxia-mediated expression of VEGF.

**Figure 6:**
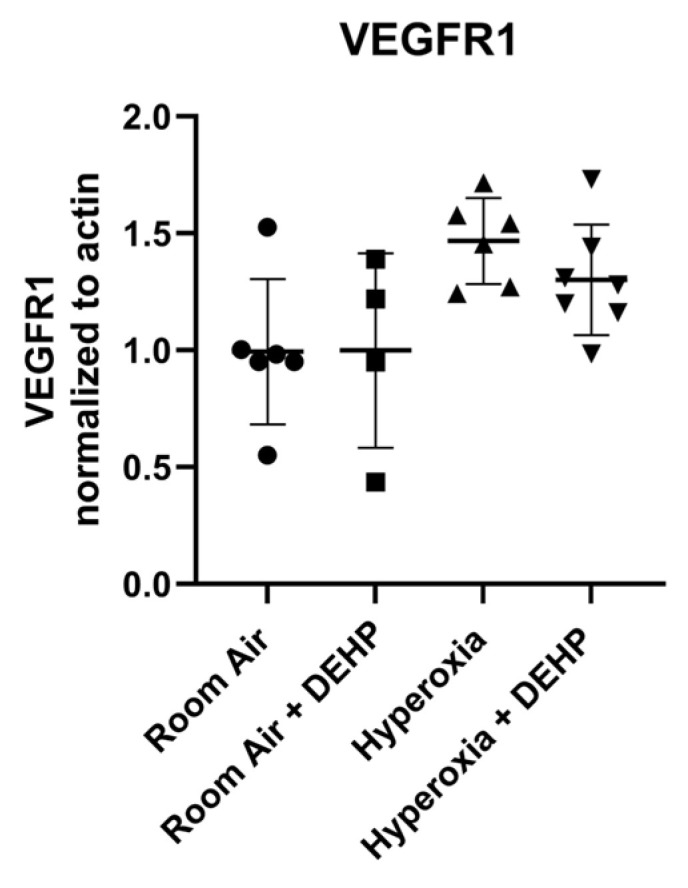
Relative abundance of VEGFR1 in lung homogenate. Neonatal Sprague Dawley rats breathing the mixture were observed for 14 days. Quantification of fluorescence intensity on immunoblots. Mean signals from each animal were used to generate Mean ± SD for each group. Data is shown as Mean ± SD. n = 4–7 animals/intervention. Significance was determined using one-way ANOVA with Tukey’s correction.

**Figure 7:**
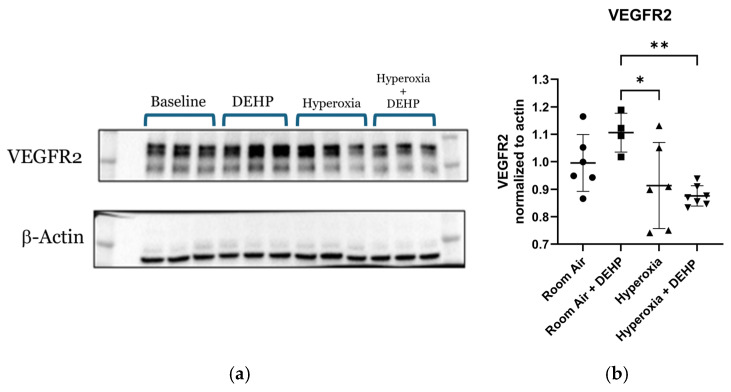
Relative abundance of VEGFR2 in lung homogenate. Neonatal Sprague Dawley rats breathing the mixture were observed for 6 h/day for 14 days. Significance was determined using one-way ANOVA with Tukey’s correction. * = *p* ≤ 0.05, ** = *p* ≤ 0.01. (**a**) Representative immunoblot. Signal generated by VEGFR2 normalized to signal generated by β-actin in the same sample. Intensity from each animal was assessed in triplicate; the mean of these was used for quantification. (**b**) Quantification of fluorescence intensity on immunoblots. Mean signals from each animal were used to generate Mean ± SD for each group. n = 4–7 animals/intervention.

The effects of VEGF are mediated by binding to two receptors, VEGFR1 and VEGFR2. We therefore examined the abundance of these receptors in our three treatment groups. The difference in VEGFR1 abundance was not statistically different between the groups (Figure 6). As detailed in Figure 7, exposure to hyperoxia, as well as combined exposure to hyperoxia + DEHP, decreases the expression of VEGR2 compared to DEHP and RA alone. However, the abundance of VEGFR2 in all three treatment groups was not statistically different from RA control group.

## 4. Discussion

Phthalate diesters are common industrial organic chemicals found in a wide array of medical equipment commonly used in NICUs [38]. These compounds have well-documented effects on endocrine responses [39,40]. Multiple epidemiologic studies have demonstrated an association between exposure during the extremely vulnerable early-life developmental window and chronic dysfunction in several organ systems [41]. To date, mechanistic information about how inhalational exposure to DEHP during the neonatal period affects lung development has been lacking. Our animal experiments show that early-life exposure to DEHP is associated with distorted alveologenesis, decreased angiogenesis and enhanced inflammatory response to oxidative stress. These findings are similar to changes found in CLD in human [3,42]. In postmortem human specimens, chronic lung disease of prematurity is characterized by (1) morphologic or histologic evidence of parenchymal injury (distorted, malformed, or absent alveoli, edematous alveolar septa), (2) local or systemic inflammation (infiltrate of polymorphonuclear [PMN] cells in the lung tissue, (3) serum markers of oxidative injury, and 4) aberrant or inadequate vascularization (low levels of expression of angiogenic growth factors) [43].

This is the first animal model to evaluate the direct effects of inhaled DEHP on early neonatal lung development, with or without hyperoxia. Both DEHP exposure alone and coincident exposure to hyperoxia and DEHP showed poor growth manifested as low body weight and histopathologic findings similar to those seen in CLD. These findings perhaps provide a key to the pathogenesis of CLD in infants who have a relatively benign NICU course.

Infants born prematurely spend a critical period of development in the chemically intensive NICU environment [13]. Phthalates are ubiquitous chemical adjuvants used to improve the flexibility and durability of common medical equipment. Phthalates, including DEHP, leach from the underlying plastic scaffold under conditions of heat and humidity [13]. Preterm infants are at high risk of toxicity due to immature pulmonary architecture [44], immature antioxidant [45] and anti-inflammatory [46] mechanisms, decreased and immature gas exchange surface area [44,47] and decreased capability to metabolize and excrete toxins [48].

It has previously been shown that DEHP exposure promotes an inflammatory lung pathology in adult mice [49]. In this study, we adapted an established animal model of prematurity-related CLD and found that exposure to DEHP in the early neonatal period leads to an inflammatory response and contributes to or exacerbates the development of CLD of prematurity. The characteristic hallmarks of CLD are a large and simplified alveolar structure, a reduced and dysmorphic vascular bed, and septal wall fibrosis [50,51]. In our model, lung injury and inflammation were confirmed by histology and immunofluorescence staining. We observed increased inflammatory markers (PMN accumulation, MPO expression), thickened septal walls possibly due to a combination of enhanced inflammatory cell infiltration and altered alveolar growth, and diminished vascularization, evidenced by lower CD31 and VEGF expression.

Exposure to hyperoxia has been demonstrated to cause impaired growth in animal models [52]. The effect of phthalate on growth is complex and depends on the timing of exposure. Infants with BPD also show impaired growth in established disease [53]. Our study shows decreased body weight in DEHP, hyperoxia-, and hyperoxia + DEHP-exposed groups compared to the RA. Reduction in body weight could also reflect generalized systemic toxicity or metabolic alterations that were not explored in our study. Body weight is an indirect measure of growth, but in the absence of birth weight and other parameters, it is difficult to suggest its association with phthalate exposure.

Histological examination of lung tissue shows scanty blood vessels with distorted alveolar growth in pups exposed to DEHP. Concomitant hyperoxia and DEHP exposure showed exacerbation of these changes and lung damage, similar to what is seen in preterm infants with CLD. Our data showed a significant decrease in RAC in the DEHP-exposed group, while coincident exposure to both hyperoxia and DEHP demonstrated a further decrease in RAC. This decrease in RAC may reflects both arrest in alveolar development and as well as structure injury to the alveoli simultaneously.

In addition to exploring histological changes, our immunoflourescence staining shows reduced expression of CD31 after exposure to DEHP, hyperoxia and Hyperoxia + DEHP. CD31 expression indicates the presence of blood vessels, as it is highly expressed at endothelial cell intercellular junctions and serves as a widely used endothelial marker in immunohistochemistry [54,55]. However, CD31 is an indirect marker and may not necessarily reflect functional or structural loss of vascular bed by itself.

We were able to measure markers of angiogenesis known to impact pulmonary development [18,35,56]. Our experiments showed that neonatal pups exposed to DEHP in early postnatal life demonstrate significantly lower expression of VEGF. As pulmonary vascular growth precedes tissue growth, lower VEGF expression may be the primary factor associated with poor alveolar growth in infants developing CLD related to DEHP exposure. This finding highlights a possible role of DEHP in lung injury in early life, even in normoxia. VEGF regulates angiogenesis by activating its receptors VEGFR1 (sFlt-1) and VEGFR2 (KDR/Flk1). VEGFR1 plays a negative role in vasculogenesis, as it does not have any mitogenic effect on endothelial cells [57]. VEGFR1 is also described as an antiangiogenic factor, as it binds extracellularly to VEGF and decreases its availability to VEGFR2 [58]. VEGFR2 is a major signal transducer for angiogenesis, promoting migration, differentiation, and proliferation of endothelial cells [59] and the formation of vascular tubes [60]. Our study shows that DEHP exposure in the early neonatal period is associated with decreased expression of VEGF without significant change in the expression of VEGFR1/VEGFR2. Thus, the effects of hyperoxia and hyperoxia + DEHP on the abundance of elements in the VEGF pathway appear to be limited to the biosynthesis of VEGF itself.

Malondialdehyde is a marker of oxidative damage that is associated with the development of CLD of prematurity [61]. Our experiments demonstrated a significant elevation of MDA levels in pups exposed to DEHP, again indicating that DEHP may contribute to oxidative damage in newborn rat pups. Interestingly, malondialdehyde levels were most pronounced in the DEHP-only exposure group, compared to those observed under hyperoxia alone or the hyperoxia + DEHP group. This finding suggests that DEHP acts as a particularly potent, and potentially primary, initiator of oxidative stress in this model, driving inflammation-mediated lung damage either through mechanisms distinct from hyperoxia or by dominating the overall oxidative burden, even when hyperoxia is also present.

Overall, our experiments demonstrate that DEHP exposure is associated with inflammatory lung damage, enhanced oxidative stress, and reduced vasculature, leading to histological changes in pulmonary architecture in a neonatal rat model. Our data support the hypothesis drawn from epidemiological studies [10,13] that DEHP exposure may be an important contributor to the development of chronic lung disease. As DEHP exposure is a potentially modifiable factor in the preterm infant hospital environment, there is an urgent need to further explore both this pathological link and the possible replacement of DEHP in common NICU respiratory circuits with less toxic chemical plasticizers.

A key feature of our animal model is that it allows for disentanglement of the role of volutrauma, barotrauma, and hyperoxia from the impact of DEHP exposure—a feat that would be virtually impossible in patient-based studies at the present time. In clinical populations, significant inhalational DEHP exposure is derived from respiratory support equipment [13], which also confers some degree of pressure and/or volume support to the developing lung. In our model, rats breathe naturally in an unsupported manner, eliminating any contribution from volutrauma or barotrauma. Newborn rat pups are at the saccular stage of lung development at birth, akin to preterm infants at the highest risk for CLD born at 24–26 weeks of gestational age [23]. They therefore provide a unique avenue for examining the developmental impact of various inhalational environmental exposures.

Our study does have limitations. Rat models do not perfectly mimic human biology; thus, there may be differences in disease progression in animals compared to humans such that our findings may not perfectly represent human pathology. Variability by sex may play a role in the biological impact of DEHP, a known endocrine disruptor associated with sexually dimorphic outcomes in humans [62]. Our study was not powered to explore sex variability in the pathogenesis of BPD. Additionally, we used high concentrations of DEHP in our experiments; although studies of preterm infant DEHP exposure indicate similarly high levels of exposure [13,63,64,65,66], it is possible that hospitalized preterm infants do not experience this level of exposure in all circumstances.

Traditionally, neonatology research has not evaluated the impact of environmental exposures on chronic lung disease development. Our study builds on the existing animal and human epidemiological literature to demonstrate the link between inhalational phthalate exposure and lung damage that is histopathologically similar to what is seen in human CLD. Human studies on the clinical impact of environmental toxicants, including DEHP, are challenging. Epidemiological observational human studies of inhalational DEHP exposure via respiratory support equipment are universally subject to confounding by indication, as the source of DEHP exposure is the respiratory support equipment that also generates barotrauma and volutrauma. Studying DEHP both as a single toxicant and as a mixture with hyperoxia is critical as both RA and oxygen therapy via the respiratory circuit are common in NICU care. Our animal model allows for isolation of these exposures and specific mixtures through the ambient free breathing environment, eliminating confounding by indication. Understanding the highly variable nature of DEHP exposure in the NICU is critical for bridging preclinical findings to clinical practice. Exposure levels can differ and clinical impact can be influenced by a confluence of factors such as gestational age, birth weight, clinical severity, type of medical devices, duration of use, and route of exposure. Characterizing this profound heterogeneity is essential for advancing translational research: it allows for the identification of at-risk patient subpopulations and provides a crucial framework for assessing whether the exposure levels employed in animal models accurately reflect the range of exposures encountered in clinical settings.

Chronic lung disease of prematurity is a complex and multifactorial disease and a central driver of the cost of care, the median hospitalization cost of a preterm infant with CLD is almost double that of an otherwise comparable preterm infant without CLD [67]. Our study highlights that DEHP, a major component of the NICU environment despite recent efforts toward exposure mitigation, may contribute to adverse pulmonary outcomes among preterm infants. Therefore, decreasing or eliminating the use of DEHP in the production of neonatal respiratory support circuits could positively impact preterm infant lung development, reduce costs of care, and reduce significant suffering in the preterm population.

## 5. Conclusions

In our novel animal model, inhaled DEHP induced lung inflammation and impaired angiogenesis and alveolarization, similar to results seen in pathological specimens taken from preterm infants with severe CLD. DEHP and hyperoxia exposure separately and together elicited morphological lung injury and inflammation characteristic of CLD.

DEHP exposure in the NICU may be toxic to the developing lungs of preterm infants and thus may be a preventable contributor to CLD. Our study was designed to identify and characterize the impact of DEHP exposure on preterm lung injury, demonstrating its significance and justifying the need for further investigation. Unraveling the complete molecular cascade is a complex endeavor that often requires a multi-stage approach. This current work serves as an essential foundation, pointing us towards the specific biological questions that now need to be addressed to develop comprehensive mitigation strategies.

## Figures and Tables

**Table 1:** Environmental exposure to experimental groups. Newborn Sprague Dawley rat pups were divided into four groups based on concentration of inhaled oxygen and/or DEHP.

Group	Number of Pups	O_2_ Concentration (%)	DEHP Concentration (mg/m^3^)
Room Air	13	21	0
Room Air + DEHP	8	21	25
Hyperoxia	14	60	0
Hyperoxia + DEHP	14	60	25

## Data Availability

The data sets generated and/or analyzed during the current study are available from the corresponding author on reasonable request.

## Abbreviations

The following abbreviations are used in this manuscript:

CLD	Chronic lung disease of prematurity
DEHP	di(2-ethylhexyl) phthalate
RA	Room Air
MPO	Myeloperoxidase
MDA	Malondialdehyde
VEGF	Vascular endothelial growth factor
BPD	Bronchopulmonary dysplasia
NICU	Neonatal Intensive Care Unit
IACUC	Institutional Animal Care and Use Committees
ARRIVE	Animal Research: Reporting of In Vivo Experiments
BSL2	Biosafety level 2
H&E	Hematoxylin and Eosin
OCT	Optimal Cutting Temperature compound
IF	Immunofluorescence
PMN	Polymorphonuclear leukocytes
RAC	Radial Alveolar Count
DAPI	4′6-diamidino-2-phenylindole
CD31/PECAM-1	Platelet Endothelial Cell Adhesion Molecule-1/CD31
cDNA	Complementary DNA
SDS-PAGE	Sodium Dodecyl Sulfate–Polyacrylamide Gel Electrophoresis
PVDF	Polyvinylidene Fluoride
IgG	Immunoglobulin G
SD	Standard Deviation
ANOVA	Analysis of Variance

## Appendix A

**Table A1:** Number of values, minimum, maximum, range, and mean of weight of rat pups in each intervention group.

	Room Air	Room Air + DEHP	Hyperoxia	Hyperoxia + DEHP
Number of values	13	8	14	14
Minimum	32.00	29.00	28.00	26.00
Maximum	40.00	31.00	33.00	31.00
Range	8.000	2.000	5.000	5.000
Mean	35.42	29.88	29.71	27.29
